# HONMF: integration analysis of multi-omics microbiome data via matrix factorization and hypergraph

**DOI:** 10.1093/bioinformatics/btad335

**Published:** 2023-05-22

**Authors:** Yuanyuan Ma, Lifang Liu, Yingjun Ma, Song Zhang

**Affiliations:** School of Computer Engineering, Hubei University of Arts and Science, Xiangyang, Hubei, China; School of Computer & Information Engineering, Anyang Normal University, Anyang, Henan, China; School of Physics and Electronic Engineering, Hubei University of Arts and Science, Hubei, China; School of Applied Mathematics, Xiamen University of Technology, Xiamen, Fujian, China; School of Computer & Information Engineering, Anyang Normal University, Anyang, Henan, China

## Abstract

**Motivation:**

The accumulation of multi-omics microbiome data provides an unprecedented opportunity to understand the diversity of bacterial, fungal, and viral components from different conditions. The changes in the composition of viruses, bacteria, and fungi communities have been associated with environments and critical illness. However, identifying and dissecting the heterogeneity of microbial samples and cross-kingdom interactions remains challenging.

**Results:**

We propose HONMF for the integrative analysis of multi-modal microbiome data, including bacterial, fungal, and viral composition profiles. HONMF enables identification of microbial samples and data visualization, and also facilitates downstream analysis, including feature selection and cross-kingdom association analysis between species. HONMF is an unsupervised method based on hypergraph induced orthogonal non-negative matrix factorization, where it assumes that latent variables are specific for each composition profile and integrates the distinct sets of latent variables through graph fusion strategy, which better tackles the distinct characteristics in bacterial, fungal, and viral microbiome. We implemented HONMF on several multi-omics microbiome datasets from different environments and tissues. The experimental results demonstrate the superior performance of HONMF in data visualization and clustering. HONMF also provides rich biological insights by implementing discriminative microbial feature selection and bacterium–fungus–virus association analysis, which improves our understanding of ecological interactions and microbial pathogenesis.

**Availability and implementation:**

The software and datasets are available at https://github.com/chonghua-1983/HONMF.

## 1 Introduction

With the rapid development of high throughput sequencing techniques, more and more microbiome data have been accumulated (Janda and Abbott 2007, Wayne Litaker *et al.* 2007, De Vries *et al.* 2012, Callahan *et al.* 2016). The bacterial, fungal, and viral microbiome can be simultaneously profiled by using different sequencing methods, such as 16S rRNA (Janda and Abbott 2007), (ITS1) rRNA (Callahan *et al.* 2016), and VIDISCA-NGS (De Vries *et al.* 2012). The multi-omics microbiome datasets generated by these technologies provides an unprecedented opportunity to understand the diversity of bacterial, fungal, and viral components (Honda and Littman 2012, Belkaid and Hand 2014). The previous studies reported that some critical illness was closely linked to changes of the composition of viruses, bacteria, and fungi (Legoff *et al.* 2017, Zuo *et al.* 2019). Dissecting the difference of microbial components from different samples is important to understand pathogenic mechanism. Computational approaches that utilize only single bacterial or viral composition profile cannot comprehensively reveal the manners that microbes play in shaping microbial ecology.

Increasing evidence also shows that there exists complicated relationship (high-order interaction) among the bacterial microbiome, viral microbiome, and host (Pfeiffer and Virgin 2016, Shkoporov and Hill 2019). These findings provide important clues that cross-kingdom interactions potentially induce diseases, but a knowledge gap remains on the manners and strength of interactions of bacteria, fungi, and viruses. Exploring the interactions between bacteria and viruses, fungi, and viruses is key to understand their latent roles in the development of inflammatory bowel disease, cancer, and sepsis (Sokol *et al.* 2017, Sovran *et al.* 2018, Haak *et al.* 2021). Hence, there is an essential need for integrative analysis frameworks that can systematically identify microbial latent patterns and associations relationships across different conditions (Richard and Sokol 2019).

Recently, unsupervised learning methods have been developed to integrate multi-omics data from the same samples, including the SNF framework and its variants (Wang *et al.* 2014, Zhang *et al.* 2017, Liu and Shang 2018), but these methods are not initially designed for microbiome data analysis. The integration methods for multi-omics microbiome data include WSNF (Mac Aogáin *et al.* 2021) and MOFA (Argelaguet *et al.* 2018, 2020, Haak *et al.* 2021). WSNF assumes that there exists a consensus sample similarity network, and it is shared across distinct composition profiles. MOFA assumes that the latent variables, i.e. low-dimensional representation of the samples, are the same for the bacterial, fungal, and viral composition profiles data. However, these assumptions may be restrictive for multi-omics microbiome data, because these compositional profiles have different characteristics. MOFA takes the three microbiome abundance matrices (bacteria, fungi, and viruses) as input, and learns a low-dimensional representation of the samples and three feature-by-factor loading matrices (one per kingdom). Compared with MOFA, one drawback of SNF and WSNF is that the similarity matrices represent the similarity between the samples, and they cannot provide direct biological insights of the microbial features.

**Figure 1.**
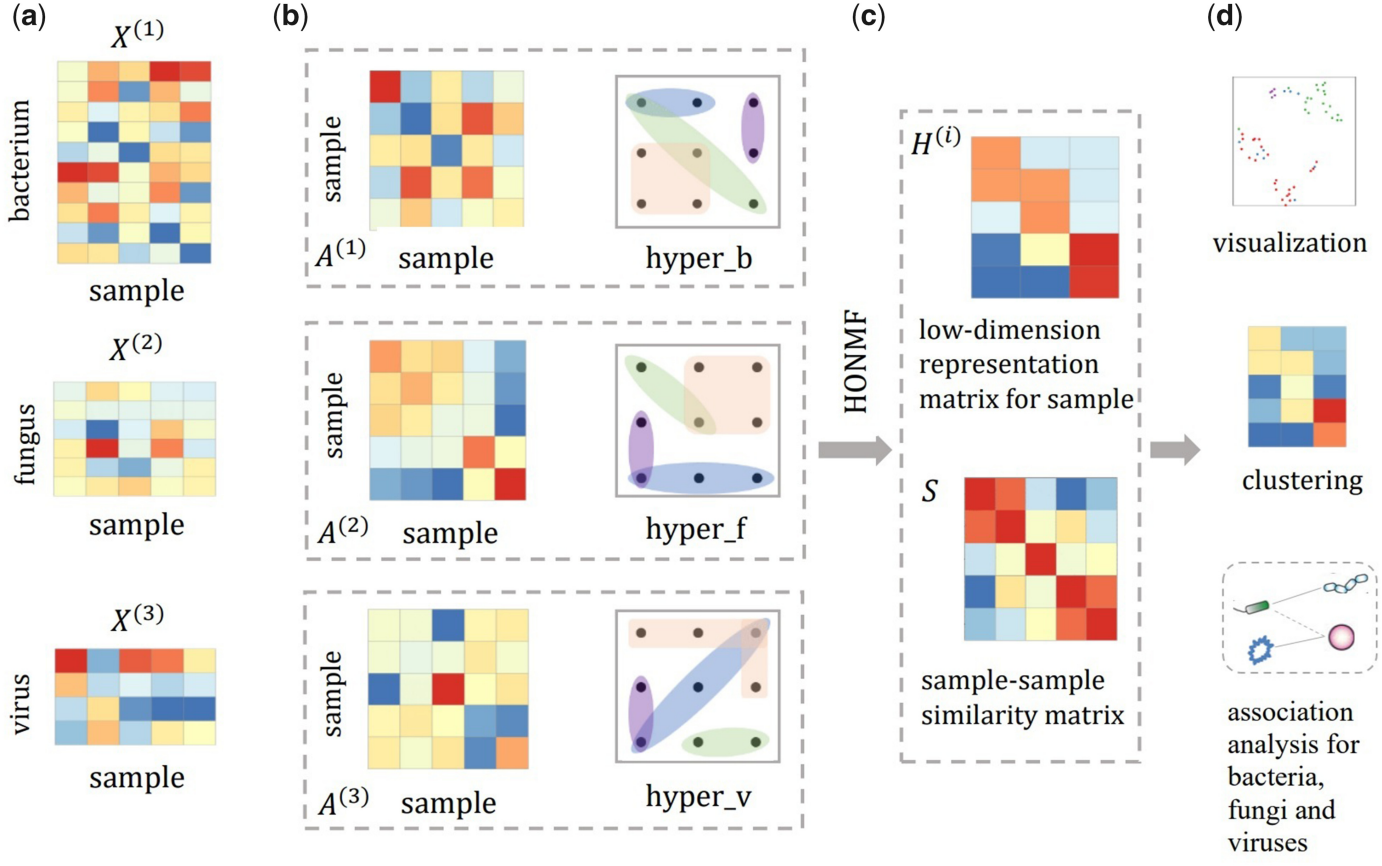
An illustrative example of HONMF. HONMF is designed for analyzing multi-omics microbiome data where bacterial, fungal and virus composition profiles data are simultaneously obtained. (a) Bacterial, fungal and virus abundance matrices $X^{\left(1\right)}$, $X^{\left(2\right)}$ and $X^{\left(3\right)}$ (each row represents a feature, e.g. bacterium, fungus or virus, and each column represents a sample). (b) For each composition profile, sample-sample similarity matrix $A^{\left(i\right)}$ is firstly computed via kernel function. Simultaneously, hypergraph is constructed based on each composition profile matrix. Then sample similarity matrices and hypergraphs are used as inputs of HONMF. (c) HONMF learns the low-dimension representation matrices of samples (i.e. the latent variables) $H^{\left(i\right)}$, and the sample-sample similarity matrix S that summarizes the information in the $H^{\left(i\right)}$, $H^{\left(2\right)}$ and $H^{\left(3\right)}$. (d) The sample-sample similarity matrix S facilitates downstream analysis, including data visualization and clustering. In addition, S can also be used to discriminative microbial feature selection and bacterium-fungus-virus associations analysis.

In this manuscript, we propose HONMF to systematically integrate multi-omics microbiome data, where bacterial, fungal, and virus compositional profiles were obtained from the same samples. HONMF is a versatile tool that enables clustering of the samples and data visualization, and it facilitates downstream biological analysis, including feature selection and cross-kingdom association analysis, such as bacterium–virus interaction, fungus–virus interaction. HONMF is a novel unsupervised learning framework, named hypergraph induced orthogonal non-negative matrix factorization (NMF). Unlike SNF and WSNF which assume that a consensus similarity network is shared across different modalities, HONMF assumes that latent variables are specific for each modality and integrates three sets of latent variables through graph fusion strategy, which better tackles the distinct characteristics in bacterial, fungal, and virus compositional profiles. In addition, HONMF preserves the high-order geometrical structures in original data by hypergraph, which is an important way to reveal the complex relationships for more than two species. Analyzing on three multi-omics microbiome datasets from different tissues (including gut and sputum) and environment (soil), we show that HONMF is effective in identifying sample types: HONMF achieves superior performance in clustering and data visualization. The learned sample–sample similarity matrix has good biological meaning: it absorbs the complementary information from each data modality, and encodes high-order interaction information from distinct composition profiles; the similarity matrix can be used to identify discriminative bacteria, fungi, or viruses in different sample clusters. HONMF can also implement bacterium–fungus–virus association analysis based on these discriminative microbial features, which improves our understanding of ecological interactions and microbial pathogenesis. An overview of HONMF is shown in Fig. 1.

## 2 Materials and methods

### 2.1 Datasets and data preprocessing

The first dataset used in this manuscript was from literature (Haak *et al.* 2021) and are downloaded from GitHub repository(https://github.com/bwhaak/MOFA_microbiome). Faecal samples from 33 patients admitted to the Intensive Care Unit (ICU) and 13 healthy individuals were collected. Of these patients, 24 were admitted with sepsis while 9 patients had a non-septic ICU diagnosis. Both bacterial 16S rRNA and the fungal ITS rDNA gene are parallelly profiled from the same single gut samples. In addition, virus composition profile is simultaneously obtained using VIDISCA-NGS.

The second dataset downloaded from NCBI SRA (PRJNA59025) included 166 patients with stable bronchiectasis. Sputum sample from each participant was collected and simultaneously sequenced (Mac Aogáin *et al.* 2021). The bacterial, fungal, and virus composition profiles were obtained after extracting sputum DNA and RNA using a standard pipeline previously described (Coughlan *et al.* 2012).

The third dataset downloaded from Wagg *et al.* (2019) was for soil ecosystems. Both bacterial and fungal profiles were simultaneously sequenced using 16S and ITS for 48 samples. Note that the virus compositional profile was not provided in the original publication, we further modified the proposed model to implement the two-modal microbiome dataset described above.

The statistical information of these three data has been presented in Supplementary Table S4.

### 2.2 Overview of NMF

Given a data matrix $X\in R_{+}^{p\times n}$, the traditional nonnegative matrix factorization (NMF) aims to find two low-rank matrices $W\in R_{+}^{p\times k}$ and $H\in R_{+}^{k\times n}$ to approximate *X*, where *p* is the number of features, *n* is the number of samples and k is the number of factors (Lee and Seung 1999). The objective function of NMF is as following. where W is basis matrix, and H is coefficient matrix, both are non-negative. ${\left\|\cdot \right\|}_{F}$ denotes Frobenius norm of a matrix. The matrix H represents the low-dimensional representation for the observations.


(1) $$\underset{W,H\geq 0}{\min}{\left\|X-WH\right\|}_{F}^{2},$$

Besides the classic NMF, tri-factor symmetric NMF (tri-sNMF) was also used for data representation and clustering (Ding *et al.* 2006, Li and Ding 2006). The objective function is defined as the following. where $H^{T}$ denotes the transpose of matrix H, *G* is a symmetric matrix and *I* is the identity matrix with suitable size. Compared with NMF, the advantages of tri-sNMF lies in: *H* is closer to the form of clustering and S provides a good indicator for clustering quality (Ding *et al.* 2005, Ma *et al.* 2020). The clusters are well separated, when the diagonal elements in *S* are much larger than the off-diagonal elements (Ding *et al.* 2005).


$$\underset{H,G\geq 0}{\min}{\left\|X-HGH^{T}\right\|}_{F}^{2},$$

(2) $$s.t.\;\;H^{T}H=I,$$

### 2.3 HONMF model

To dissect microbial sample heterogeneous from bacterial, fungal and virus composition profile level, we introduce hypergraph induced orthogonal nonnegative matrix factorization model (HONMF). Given the bacterial composition profile matrix $X^{\left(1\right)}\in R_{+}^{p\times n}$ (p bacteria species in n samples), the fungal profile matrix $X^{\left(2\right)}\in R_{+}^{q\times n}$ (q fungal species in n samples) obtained from the same samples, and the virus profile matrix $X^{\left(3\right)}\in R_{+}^{r\times n}$ (r viral pathogens in n samples), HONMF aims to learn a consensus sample-sample similarity matrix $S\in R_{+}^{n\times n}$, which integrates multiple molecular modalities obtained from the same microbial samples. The objective function of HONMF is as the following:


$$\underset{{H^{\left(i\right)},G}^{\left(i\right)},S}{\min}\;J=\sum_{i=1}^{3}{\left\|A^{\left(i\right)}-H^{\left(i\right)}{G^{\left(i\right)}H}^{{\left(i\right)}^{T}}\right\|}_{F}^{2}+\frac{\alpha }{2}\sum_{i=1}^{3}{\left\|S-H^{\left(i\right)}H^{{\left(i\right)}^{T}}\right\|}_{F}^{2}+\eta \sum_{i=1}^{3}{\left\|H^{{\left(i\right)}^{T}}H^{\left(i\right)}-I\right\|}_{F}^{2}+\beta {\left\|S1-1\right\|}_{F}^{2}+\gamma \sum_{i=1}^{3}tr\left(H^{{\left(i\right)}^{T}}L_{\mathrm{hyg}}^{\left(i\right)}H^{\left(i\right)}\right)$$

(3) $$s.t.\;{\;H}^{\left(i\right)},G^{\left(i\right)},S,\alpha ,\beta ,\gamma ,\eta \;\geq 0.$$

Here, $H^{\left(i\right)}\in R_{+}^{n\times k}$ represents the matrix of low-dimensional representation (i.e., latent variables) for the ith data modality (bacterial, fungal or virus composition profile) of the samples. $G\in R_{+}^{k\times k}$ represents the connections among different clusters and is symmetric. *S* is the learned sample-sample similarity matrix that can be used for clustering and data visualization of the microbial samples. 1 is a column vector with all elements to be 1s. $L_{\mathrm{hyg}}^{\left(i\right)}$ represents the hypergraph Laplacian for the ith data modality, and it captures high-order relationship in original microbiome data (Zhou *et al.* 2006, Gaudelet *et al.* 2018, Jin *et al.* 2019). $A^{\left(i\right)}$ is the similarity matrix obtained from ith microbial composition profile matrix $X^{\left(i\right)}$. η is the parameter that reflects strength of the orthogonal constraint imposed to the columns of $H^{\left(i\right)}$, and is set η=10 for all datasets. β is a parameter that is used to control the strength of the constraint S1=1 and is set β=1 for all datasets. α is a parameter used to control the strength of regularization that makes each kernel $H^{\left(i\right)}H^{{\left(i\right)}^{T}}$ from each composition data towards a consensus graph S. Large values of α imply more closer between $H^{\left(i\right)}H^{{\left(i\right)}^{T}}$ and S. γ is graph regularization parameter. In the later section, we will discuss how to choose α and γ.

In the objective function of HONMF [Equation (3)], the first term, $\sum_{i=1}^{3}{\left\|A^{\left(i\right)}-H^{\left(i\right)}{G^{\left(i\right)}H}^{{\left(i\right)}^{T}}\right\|}_{F}^{2}$, is standard tri-factor symmetric NMF loss function for bacterial, fungal and virus composition profile data. The sample similarity matrix $A^{\left(i\right)}$ can be obtained by Gaussion kernel function. The details of constructing $A^{\left(i\right)}$ are presented in Additional file 1. The second term, $\sum_{i=1}^{3}{\left\|S-H^{\left(i\right)}H^{{\left(i\right)}^{T}}\right\|}_{F}^{2}$, is a consensus graph fusion operation that integrates different composition profile data to learn a sample-sample similarity matrix S. One of the advantages is that it regularizes each kernel $H^{\left(i\right)}H^{{\left(i\right)}^{T}}$ from each composition data towards a consensus graph S. The third term, $\sum_{i=1}^{3}{\left\|H^{{\left(i\right)}^{T}}H^{\left(i\right)}-I\right\|}_{F}^{2}$, encourages the low-dimensional representations $H^{\left(i\right)}$ to be column-orthogonal, and is used to preserve the uniqueness of the solution. The fourth term, ${\left\|S1-1\right\|}_{F}^{2}$ is a normalization term on S that encourages each row in S to have summation close to 1.

Through iteration fusion, the first four terms in Equation (3) can learn the low-dimensional representation for each data modality, however, high-order interaction relationships involving more than two species may be lost. Some literatures reported that high-order microbial interactions are prevalent and dominate the functional landscape of microbial communities, and enable bacteria to deal with new complex environments (Sanchez-Gorostiaga *et al.* 2019, Ludington 2022). However, graph-based learning methods, such as graph Laplacian, only consider pairwise interaction (Cai *et al.* 2010, Ma *et al.* 2020). Hypergraph can effectively solve this problem. Unlike classic graph in which two vertices are linked by an edge, a group of vertices is viewed as a hyperedge in a hypergraph (Zhou *et al.* 2006). Modeling these high-order interactions with hypergraph can significantly enhance clustering performance. Therefore, we include a fifth term, $\sum_{i=1}^{3}tr\left(H^{{\left(i\right)}^{T}}L_{\mathrm{hyg}}^{\left(i\right)}H^{\left(i\right)}\right)$, in HONMF model. The details of constructing hypergraph Laplacian $L_{\mathrm{hyg}}^{\left(i\right)}$ are presented in later section.

We note that MOFA(Argelaguet *et al.* 2018, 2020) also integrates multi-modal microbiome data. Our proposed HONMF model differs from MOFA in the following several aspects: (i) MOFA assumes that *H* is shared by bacterial, fungal and virus composition profile matrices, which may be not appropriate due to batch effects. In HONMF we relax this assumption by allowing *H* to vary for each composition profile data and use *S* derived from graph fusion operation to integrate cross-modality information; (ii) HONMF adopts tri-factor NMF and encourages *H* to be column-orthogonal, and MOFA does not. tri-Factor NMF has more flexible ability in data analysis tasks (Ding *et al.* 2006). The orthogonal constraint on the low-dimensional representations of microbial samples leads to better clustering solutions and interpretability: the columns in $H^{\left(i\right)}$ will tend to be sparse; (iii) HONMF includes an additional term with hypergraph Laplacian to explore the complicated high-order microbial interactions, and simultaneously enhances the representation ability of low-dimensional factor matrices. The detailed optimization algorithm for HONMF is presented in Supplementary File S1.

### 2.4 Construction of hypergraph

In a simple graph, an edge only connects to two vertices and the edge weight indicates the relationships between these two vertices. However, in many real-world tasks, representing a set of complex relational objects as a simple graph may cause information loss. For example, to group a number of articles into corresponding topics, one can construct a simple graph where two articles are connected with an edge if there is at least one common author writes them, and then spectral clustering technique is applied (Zhou *et al.* 2006). The above graph representation method obviously misses some useful information in this case that the same author may write three or more articles. Such unexpected lost information is useful to cluster different topics.

**Figure 2.**
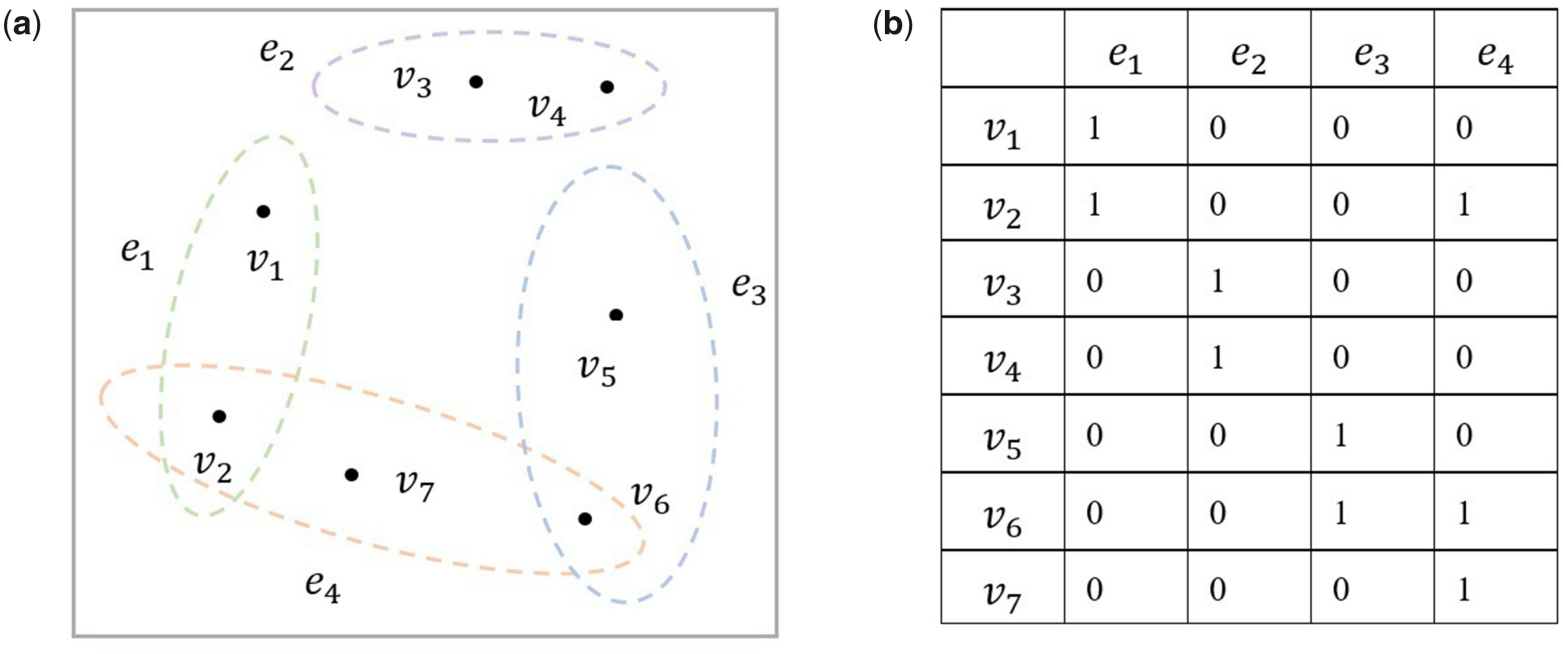
The organization of nodes and edges in hypergraph. (a) An illustrative example of a hypergraph. Here, $v_{i}$ denotes vertex, $e_{j}$ denotes hyperedge. A hyperedge can connect more than two vertices. (b) The incidence matrix corresponding to hypergraph. The entry ($v_{i}$, $e_{j}$) is set to be 1 when $v_{i}$ belongs to $e_{j}$, and 0 otherwise.

A natural way to handle with information loss problem existed in simple graph is to represent the data relationships as hypergraph (Fig. 2) In hypergraph an edge can connect more than two vertices. Let *V* denote the set of vertices and *E* denote hyperedge set. For each hyperedge *e*, $\cup_{e\in E}=V$ and its weight is denoted as $w\left(e\right)$. A weighted hypergraph is represented as $G=\left(V,E,W\right)$. *W* is a diagonal matrix which represents the weights of hyperedges. The weight construction rule for each hyperedge *e* is described as following.

The weight for hyperedge *e* is set with gaussian kernel function. Specifically, we first compute the similarity between any two nodes belonging to *e* with gaussian kernel function. Then, the total similarity is computed as the weight of hyperedge *e*. Here, the bandwidth parameter is set as the mean of the squared Euclidean distance between two nodes in the hyperedge *e*. The details are presented in Supplementary File S1.

The incidence matrix $P\in R^{\left|V\right|\times \left|E\right|}$ of *G* with entries $p\left(v,e\right)$ is defined as the following. where $\left|V\right|$ represents number of vertices in hypergraph, and $\left|E\right|$ represents number of hyperedges. For a vertex v∈V, its degree is defined as $d\left(v\right)=\sum_{e\in E}w\left(e\right)p\left(v,e\right)$. The degree of a hyperedge is defined as $\delta \left(e\right)=\sum_{v\in V}p\left(v,e\right)$. Let $D_{v}$ and $D_{e}$ denote degree matrices whose elements are the vertex degrees and hyperedge degrees, respectively. Then the Laplacian matrix of a hypergraph $L_{\mathrm{hyg}}$ can be defined as the following.


(4) pv,e=1,  if v∈e,0,  if v∉e.

(5) $$L_{\mathrm{hyg}}=D_{v}-PWD_{e}^{-1}P^{T}.\;$$

In contrast to *k-*nearest neighbors (KNN), in this manuscript we use Louvain community detection algorithm (Blondel *et al.* 2008) to construct hyperedges: each cluster is represented as a hyperedge. This strategy avoids to the influenced of outlies and leads to better interpretability.

The hypergraph captures the high-order interactions, the regularization term, i.e. the fifth term of Equation (3), can be derived by: where *H* is the low-dimensional representation of microbial samples.


(6) $$O\left(H\right)=\frac{1}{2}\sum_{e\in E}\sum_{\left(i,j\right)\in e}\frac{w\left(e\right)}{\delta \left(e\right)}{\left\|H_{i}-H_{j}\right\|}_{F}^{2}=Tr\left(H^{T}L_{\mathrm{hyg}}H\right).$$

### 2.5 Selection of parameters α  and γ

In HONMF, α and γ are the graph regularization parameters, and they are determined as the following. First, we solve the optimization problems ${\left\|A^{\left(1\right)}-H^{\left(1\right)}H^{{\left(1\right)}^{T}}\right\|}_{F}^{2}$, ${\left\|A^{\left(2\right)}-H^{\left(2\right)}H^{{\left(2\right)}^{T}}\right\|}_{F}^{2}$ and ${\left\|A^{\left(3\right)}-H^{\left(3\right)}H^{{\left(3\right)}^{T}}\right\|}_{F}^{2}$ by using NNDSVD (Boutsidis and Gallopoulos 2008) and obtain the initial solutions ${\hat{H}}^{\left(1\right)}$, ${\hat{H}}^{\left(2\right)}$ and ${\hat{H}}^{\left(3\right)}$. Second, the initial values of $G^{\left(1\right)}$, $G^{\left(2\right)}$ and $G^{\left(3\right)}$ are set as ${\hat{G}}^{\left(1\right)}$=${\hat{G}}^{\left(2\right)}={\hat{G}}^{\left(3\right)}=I$, and then we use SNF (Wang *et al.* 2014) to obtain $\hat{S}$. Finally, α and γ are set as:


(7) $$\alpha =\sum_{i=1}^{3}{\left\|X^{\left(i\right)}-{\hat{H}}^{\left(i\right)}{\hat{G}}^{\left(i\right)}{\hat{H}}^{{\left(i\right)}^{T}}\right\|}_{F}^{2}/\left(\sum_{i=1}^{3}{\left\|\hat{S}-{\hat{H}}^{\left(i\right)}{\hat{H}}^{{\left(i\right)}^{T}}\right\|}_{F}^{2}\right),$$

(8) $$\gamma =\sum_{i=1}^{3}{\left\|X^{\left(i\right)}-{\hat{H}}^{\left(i\right)}{\hat{G}}^{\left(i\right)}{\hat{H}}^{{\left(i\right)}^{T}}\right\|}_{F}^{2}/\left(\sum_{i=1}^{3}tr\left({\hat{H}}^{{\left(i\right)}^{T}}L_{\mathrm{hyp}}^{\left(i\right)}{\hat{H}}^{\left(i\right)}\right)\right).$$

### 2.6 Evaluation metrics

Normalized mutual information (NMI) (Strehl and Ghosh 2002, Vinh *et al.* 2010), adjusted rand index (ARI) (Santos and Embrechts 2009), and silhouette coefficient (Kaufman and Rousseeuw 2009) are used to evaluate the performance of the clustering methods.

Let G denote ground-truth labels of microbial samples provided in the original publications, and P denote the predicted clustering assignments. NMI is computed as the following: where $MI\left(G,P\right)\;$is the mutual information between two label sets: G and P, $H\left(G\right)\;$ and $H\left(P\right)\;$are the information entropy of G and P, respectively. High NMI values indicate good clustering consistency.


(9) $$\mathrm{NMI}\left(G,P\right)=\frac{MI\left(G,P\right)}{\sqrt{H\left(G\right)H\left(P\right)}}.$$

Assume that *N* is the number of microbial samples in a given dataset, $N_{i}$ is the number of samples in the *i*th sample types in partition *G*, $N_{j}$ is the number of samples in the *j*th cluster in partition *P*, and $N_{ij}$ is the number of samples of the *i*th label assigned to the *j*th cluster in partition *P*. ARI is defined as:


(10) ARIG,P=∑ijNij2-∑iNi2∑jNj2/N212∑iNi2+∑jNj2-∑iNi2∑jNj2/N2

For unlabeled dataset, we use an unsupervised metric, silhouette coefficient (Rousseeuw 1987, Xu *et al.* 2017), to evaluate the clustering performance. Let $a\left(i\right)$ denote the average distance of microbial sample i to all other samples within the same cluster with i, and $b\left(i\right)$ denote the average distance of i to all samples to the neighboring cluster, i.e., the smallest average distance to the cluster of i. The silhouette coefficient for microbial sample i is defined as:


(11) si=1-aibi,       if ai<bi0,               if ai=bibiai-1,    if ai>bi.

A larger silhouette coefficient of one microbial sample indicates that the sample is close to other samples in the same cluster, and distant from samples in other clusters. The average value of silhouette coefficients for all the microbial samples is computed as the final evaluation metric.

### 2.7 Identifying discriminative bacteria, fungi, or viruses with Laplacian score

Given *S* obtained from HONMF, its corresponding degree matrix $D=\sum_{i=1}S_{ij}$ and Laplacian matrix L=D-S, the Laplacian score (He *et al.* 2005) of a feature ***f*** is computed as follows.


(12) $$SC\left(f\right)=\frac{{\tilde{f}}^{T}L\tilde{f}}{\tilde{f}D\tilde{f}}$$

(13) $$\tilde{f}=f-\frac{f^{T}D1}{1^{T}D1}1.$$

Here, **1** is a column vector with all elements to be 1 s. The top *k* features with have the minimal *SC* values are picked up, and used as the downstream analysis.

## 3 Results

### 3.1 HONMF achieves good clustering performance on different datasets from different tissues and environments

We evaluated the proposed HONMF on three multi-modal microbiome datasets. These datasets include a gut microbiome dataset and a sputum dataset from patients with stable bronchiectasis, where the bacterial, fungal, and virus composition profiles were sequenced for the same sample; soil microbiome dataset, where the bacterial and fungal composition were profiled for the same sample from grassland ecosystems.

We compared HONMF with several recently published methods for multi-modal microbiome data integration, including MOFA+ (Argelaguet *et al.* 2020), SNF (Wang *et al.* 2014), and WSNF (Mac Aogáin *et al.* 2021). For SNF and WSNF, we implemented their default clustering method and all the parameters were set to default. MOFA+ gives the low-dimensional representations of the samples, and no clustering method was provided. To facilitate direct comparison, we used SNN (shared nearest neighbor graph) + Louvain clustering (Blondel *et al.* 2008) (the SNN graph was constructed by the low-dimensional matrix *Z* obtained from MOFA+) to evaluate its performance. For HONMF, we first constructed k-nearest neighbor (KNN) graph with k=n/2 (n is the number of samples) using the sample-sample similarity matrix, and then implemented Louvain clustering on the KNN graph.

**Figure 3.**
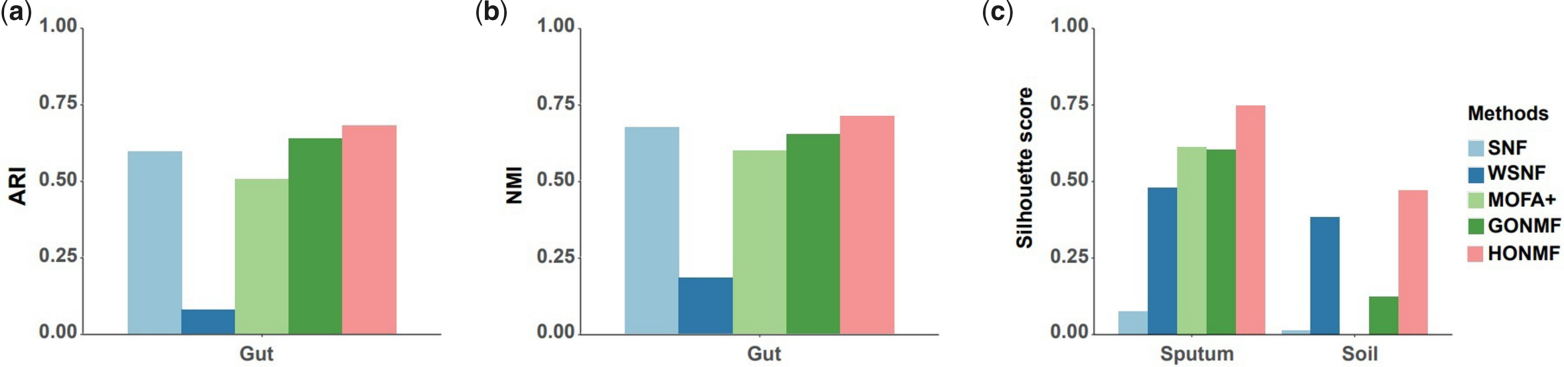
Assessment of the clustering performance. (a) Evaluation of the clustering results in terms of ARI. (b) Evaluation of clustering results in terms of NMI. The microbiome sample labels provided in the original publication were used as ground-truth labels. (c) Evaluation of the clustering results in terms of the average silhouette score. The silhouette score quantifies how well a sample is matched to its identified cluster compared to its neighboring cluster. Silhouette scores are computed based on the sample similarity matrices and labels identified by each method. Finally, the average silhouette score of all samples was reported

The clustering performance evaluated by ARI, NMI, and silhouette scores are presented in Fig. 3. For Dataset 1, ARI and NMI were computed based on the ground-truth microbial sample labels provided in the original publication. Acknowledging that silhouette score is an unsupervised metric and it does not require true labels, we also evaluated the clustering performance in terms of silhouette score.

As shown in Fig. 3, we can see that the proposed HONMF method performs well on three datasets in terms of ARI, NMI, and average silhouette score. SNF also performs well on the gut microbiome dataset in terms of ARI, but not as well as NMI. Silhouette score was computed based on the sample similarity matrix and clustering results given by each method, and it does not require “ground truth” labels. For silhouette criterion, HONMF achieves the best performance, compared with other methods. For the sputum dataset, MOFA+ performs well in the average silhouette score metric on sputum data. The numeric values of the clustering performance are presented in Supplementary Table S1.

### 3.2 Ablation study

We implemented several simple versions of HONMF [Equation (3)], where we set α,γ and η equal to 0 in turn. HONMF achieves the consistent good performance in most of the datasets (Additional file 1: Supplement Table S2). We also tested a simple variant of model (3): in GONMF, we replace hypergraph Laplacian with simple graph Laplacian. The performance of GONMF was not as good as HONMF, which suggests that it is beneficial to integrate high-order interaction information into model (3) with hypergraph (Additional file 1: Supplementary Table S3). More details for implementing are presented in Additional file 1.

### 3.3 HONMF facilitates microbiome data visualization

**Figure 4.**
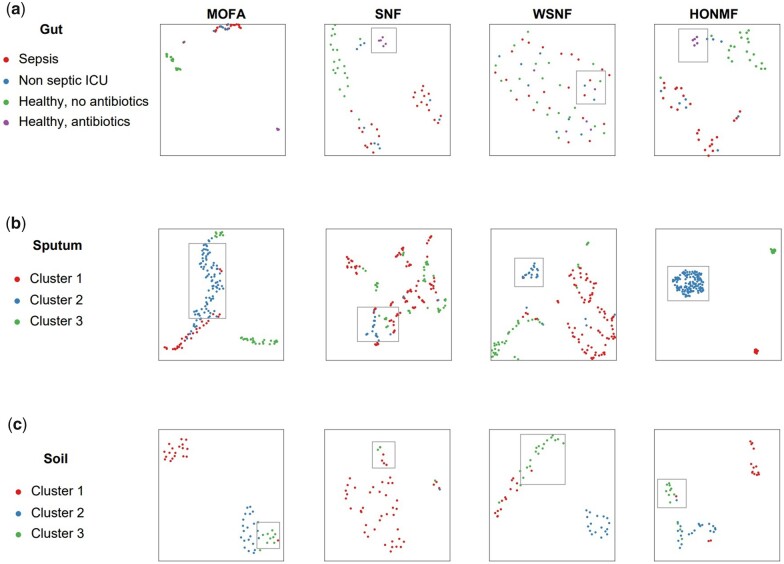
Evaluation of the visualizations of microbial samples. MOFA+, SNF, WSNF, and HONMF are compared by implementing UMAP visualization on (a) the gut microbiome dataset (bacterial + fungal +virus), (b) the sputum microbiome data with stable bronchiectasis (bacterial + fungal +virus), and (c) the soil microbiome dataset (bacterial + fungal). Samples are colored based on their labels provided in the original publications of the dataset (a) or identified clusters by each method (b, c)

We next performed UMAP visualization (McInnes *et al.* 2018) based on learned sample–sample similarity matrices and low-dimensional factor matrix. The visualization results are presented in Fig. 4. For gut data, we used the microbiome sample labels provided in its original publication for fair comparison. For sputum and soil data, we used the labels identified by each method to assess visualization results. For all three datasets and for UMAP visualization, HONMF performs well among these four methods.

As shown in Fig. 4, HONMF can also identify the less abundant sample subpopulations. The healthy individuals that received oral broad-spectrum antibiotics are clearly distinguished by HONMF. The healthy individuals (green) that did not receive antibiotics are well separated with individuals treated with antibiotics (Fig. 4a). For sputum and soil datasets, HONMF achieves the consistent good performance (Fig. 4b and c).

### 3.4 Testing clustering significance with SigClust

**Figure 5.**
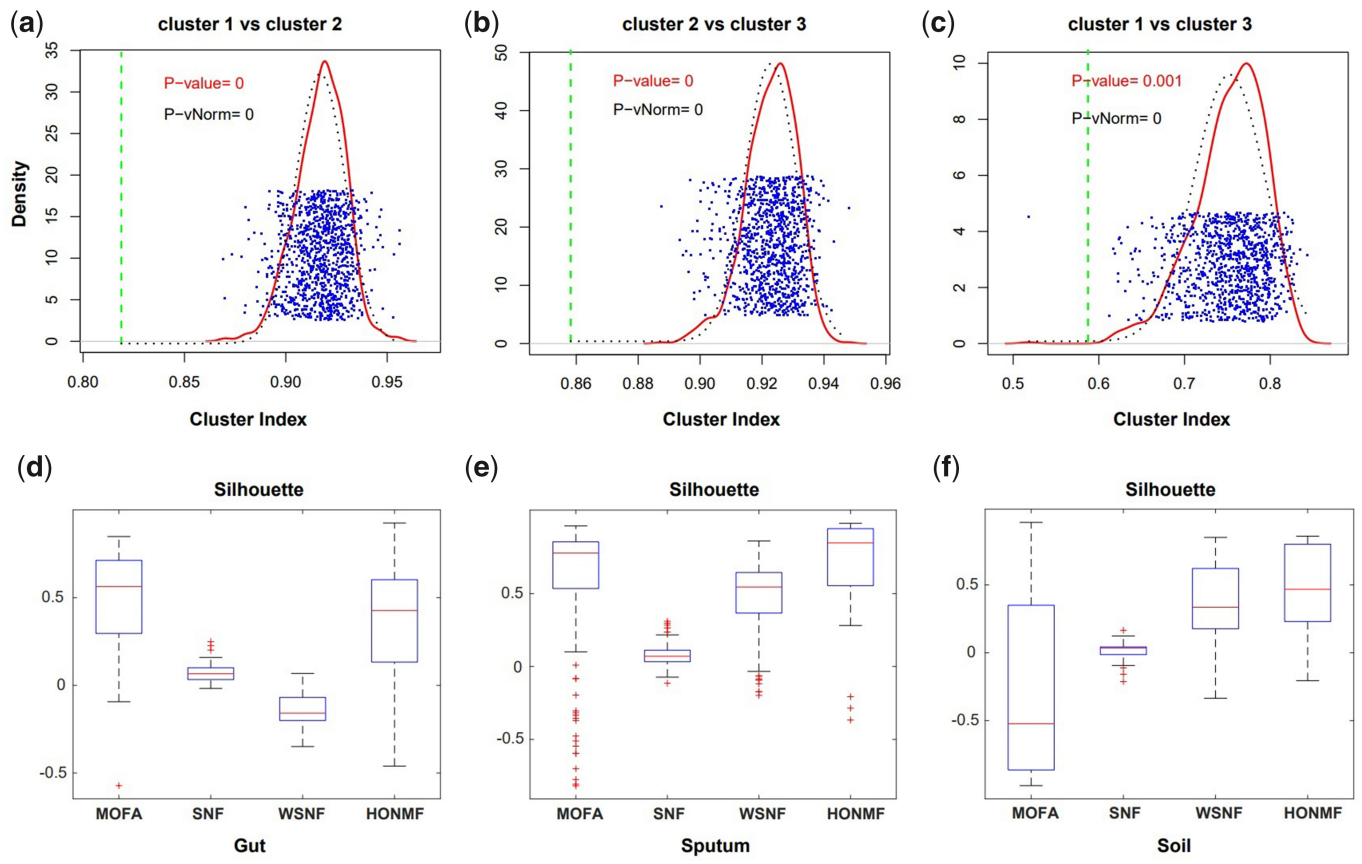
Assessment of clustering significance on gut microbiome data. (a–c) The blue points represent the simulated CIs (cluster indices). The red solid line and black dotted line correspond to the estimated nonparametric density and Gaussian density fit to the simulated CIs. (d–f) Silhouette scores are computed based on the sample-sample similarity matrices and labels obtained in each method.

To test the significance of clustering results, SigClust tool is used on these multi-omics microbiome datasets (Liu *et al.* 2008). Figure 5 shows that any two clusters obtained from HONMF are statistically significant (gut microbiome data). Statistical significance of clustering on other datasets is presented in Supplementary Fig. S1.

As shown in Fig. 5, the clustering obtained from HONMF is statistically significant. P-values between any two clusters are small or approximate to zero. The analysis of clustering significance on sputum dataset has similar results. For the clustering performance evaluated by silhouette score, HONMF is either the best or the second best among all the methods.

### 3.5 Identifying bacterium–fungus–virus associations with LS

Fungus and viruses often directly and indirectly interact with bacteria in human disease. What is known on these associations improves our understanding of ecological interactions and microbial pathogenesis. In this subsection, feature selection is firstly conducted to identify discriminative bacteria, fungi, or viruses in different groups. Then, bacterium–fungus–virus associations analysis is implemented based on these features.

For multi-omics microbiome data, some microbes play important roles in process of disease development. To identify these biological meaning microbial features, we used LS to implement feature selection (He *et al.* 2005).

Next, we implement bacterium–fungus–virus association analysis with sample–sample similarity matrix *S* obtained from HONMF and the selected features above. For microbial feature vectors *a* and *b*, their correlation is computed as follows.


(14) $$\mathrm{corr}\left(a,b\right)=\frac{\sum \left(\hat{a}-\bar{\hat{a}}\right)\left(\hat{b}-\bar{\hat{b}}\right)}{\sqrt{\sum {\left(\hat{a}-\bar{\hat{a}}\right)}^{2}}\sqrt{\sum {\left(\hat{b}-\bar{\hat{b}}\right)}^{2}}}.$$

(15) $$\hat{a}=a*S,\;\hat{b}=b*S.$$

**Figure 6.**
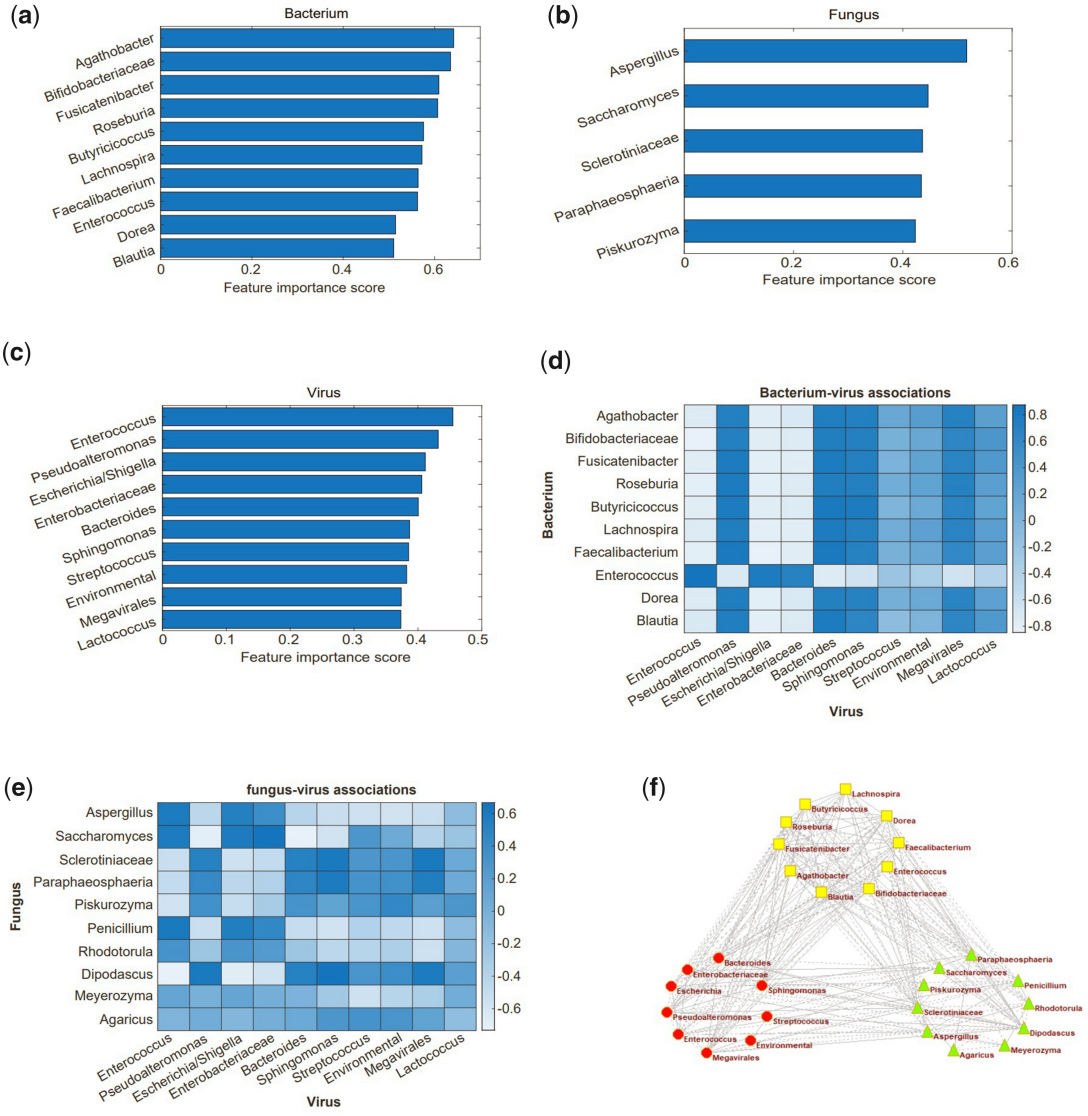
Microbial feature importance ranks and association analysis with HONMF on gut dataset. (a) Bacterium importance ranks (top 10) on faecal samples from sepsis patients and healthy individuals. LS are used to determine the importance level of microbial features. (b) Fungus important ranks. (c) Virus important ranks. (d) The heatmap of bacterium–virus interaction matrix. Bacterium–virus associations are computed by using the correlation coefficients between discriminative bacterial and viral species. (e) The heatmap of fungus–virus interaction matrix. Fungus–virus association are computed by using correlation coefficients between the discriminative fungal and viral species. (f) The bacterium–fungus–virus association network. Based on the discriminative features and sample-sample similarity matrix *S*, the correlations between them are calculated. The absolute values of correlation coefficients >0.5 are retained. The solid line indicates positive correlation, and dashed line indicates negative correlation. The yellow box denotes bacterium, the light blue triangle denotes fungus, and the red ellipse denotes virus.

Large corr(a, b) values indicate that these two features may have latent association. Figure 6 shows the discriminative bacterium, fungus, and virus features, and the associations between them.

Top 10 bacterial taxa identified by LS, including *Blautia*, *Agathobacter*, *Enterococcus*, *Roseburia*, *Lachnospira*, and *Faecalibacterium* are key features in distinguishing health and sepsis illness (Fig. 6a). These signatures were driven by antibiotic perturbation. Interestingly, some facultative aerobic bacterial pathobionts, such as *Enterococcus* have been previously associated with sepsis (Alverdy and Krezalek 2017), and bacterial taxa, *Lachnospira*, has been reported to be biomarkers of a healthy microbiota and is related to colonization resistance against bacterial pathobionts (Lee *et al.* 2017). Top five fungal taxa identified by LS, such as *Aspergillus*, *Saccharomyces*, *Paraphaeosphaeria*, and *Piskurozyma* are related to sepsis (Fig. 6b). *Piskurozyma* was previously found to be absent in critically ill patients and present in healthy subjects (Haak *et al.* 2021). For viral taxa, most important signatures are also identified by microbiome sample similarity matrix obtained from HONMF, such as *Enterococcus*, *Escherichia*, *Enterobacteriaceae*, *Bacteroides*, *Streptococcus*, and *Lactococcus* (Fig. 6c). These findings are consistent with the previous research (Haak *et al.* 2021).

Other than discriminative microbial features, the sample similarity matrix *S* facilitates bacterium–fungus–virus association analysis, which provides rich insights into microbial pathogenesis. *Blautia* and *Roseburia*, members of the Lachnospiraceag family, show negative correlations with *Saccharomyces cerevisiae* (Supplementary Fig. S4). These findings are supported by previous studies (Nguyen *et al.* 2011, García *et al.* 2017). For bacterium–virus associations, we found that *Enterobacteriaceae* has strong negative correlations with bacterium taxa *Agathobacter*, *Roseburia*, *Faecalibacterium*, *Blautia*, and *Lachnospira* (Fig. 6d). For fungus–virus associations, *Enterobacteriaceae* are positively associated with fungal taxa *Aspergillus*, *Penicillium* and *Saccharomyces*, and negatively associated with *Dipodascus* (Fig. 6e). These results are accord with Haak’s research where they use MOFA to implement factor analysis (Haak *et al.* 2021).

To summarize, HONMF facilitates the identification of discriminative features on multi-omics microbiome data, by inspecting LS scores. The association analyses for bacterium, fungus, and virus provide further indications that the gut trans-kingdom features identified by HONMF are biological meaningful.

## 4 Discussion and conclusions

The accumulation of multi-omics microbiome data provides an unprecedented opportunity to understand the diversity of bacterial, fungal, and viral components. Here, we proposed HONMF, which integrates different composition profiles in multi-modal microbiome data. Network fusion-based methods (including SNF and WSNF) typically assume that a consensus similarity network is shared across different modalities. Unlike these approaches, HONMF assumes that each composition profile has specific latent variables, and merges different sets of latent variables with graph fusion strategy (the second term in the objective function of HONMF). We conduct experiments on three multi-modal microbiome datasets. The results demonstrate that HONMF has better clustering performance and visualization, through relaxing the consensus similarity network assumption, which introduces more flexibility and better deals with the distinct characteristics in various composition profiles. HONMF also takes advantage of hypergraph learning to encode high-order geometrical structures in original data. Compared to simple graph, hypergraph leads to improved clustering qualities (Supplementary Table S3). In addition, HONMF facilitates downstream biological analysis, including microbial signature selection and cross-kingdom association analysis of gut microbiome.

In the experiments, the number of clusters *k* is set to be the one provided in the original publication: k=4 for gut dataset; k=3 for sputum and soil data. We also tested the robustness of HONMF on the number of on the number of factors (the dimension of latent features of H), where we varied the number of factors from 2 to 5. The clustering performance evaluated by NMI, ARI, and silhouette score are presented in Additional file 1: Supplementary Table S5. For sputum datasets, the performance is robust to the number of factors. The dataset that seems less robust is the gut and soil dataset. One of possible reasons is that the gut and soil microbiome data tends to have high level of noise and more complicated compositions.

For hyperparameters selection, two rules are designed to assign initial values to graph regularization parameters α and γ. To validate the effectiveness of objective function, we implement several simple versions of HONMF, where we set α,γ and η equal to 0 in turn. The experimental results show that HONMF achieves the consistent good performance (Additional file 1: Supplement Table S2). Moreover, we also implement comparison tests where simple graph Laplacian is substituted for hypergraph Laplacian. However, in most cases, the performance of GONMF was not as good as HONMF (Additional file 1: Supplementary Table S3).

The future directions of HONMF framework lie in: (i) extending HONMF to more modalities. Although HONMF integrates the abundance profiles of bacteria, fungi, and virus, it is straightforward to extend HONMF to other tasks with more than three modalities, such as Paired-Tag data (Zhu *et al.* 2021). (ii) Inferring bacteria–fungi–virus interaction relationships by introducing covariates into this framework, such as antibiotic perturbation, diet, and lifestyles (Hsiao *et al.* 2018, Haak *et al.* 2021).

## Supplementary Material

btad335_Supplementary_Data

## References

[btad335-B1] Alverdy JC , Krezalek MA. Collapse of the microbiome, emergence of the pathobiome and the immunopathology of sepsis. Crit Care Med 2017;45:337–47. 28098630 10.1097/CCM.0000000000002172 PMC5245179

[btad335-B2] Argelaguet R , Arnol D, Bredikhin D et al MOFA+: a statistical framework for comprehensive integration of multi-modal single-cell data. Genome Biol 2020;21:1–17. 10.1186/s13059-020-02015-1 PMC7212577 32393329

[btad335-B3] Argelaguet R , Velten B, Arnol D et al Multi‐omics factor analysis—a framework for unsupervised integration of multi‐omics data sets. Mol Syst Biol 2018;14:e8124. 29925568 10.15252/msb.20178124 PMC6010767

[btad335-B4] Belkaid Y , Hand TW. Role of the microbiota in immunity and inflammation. Cell 2014;157:121–41. 24679531 10.1016/j.cell.2014.03.011 PMC4056765

[btad335-B5] Blondel VD , Guillaume J-L, Lambiotte R et al Fast unfolding of communities in large networks. J Stat Mech 2008;2008:P10008.

[btad335-B6] Boutsidis C , Gallopoulos E. SVD based initialization: a head start for nonnegative matrix factorization. Pattern Recog 2008;41:1350–62.

[btad335-B7] Cai D , He X, Han J et al Graph regularized nonnegative matrix factorization for data representation. IEEE Trans Pattern Anal Mach Intell 2010;33:1548–60. 21173440 10.1109/TPAMI.2010.231

[btad335-B8] Callahan BJ , McMurdie PJ, Rosen MJ et al DADA2: high-resolution sample inference from illumina amplicon data. Nat Methods 2016;13:581–3. 27214047 10.1038/nmeth.3869 PMC4927377

[btad335-B9] Coughlan CA , Chotirmall SH, Renwick J et al The effect of *Aspergillus fumigatus* infection on vitamin D receptor expression in cystic fibrosis. Am J Respir Crit Care Med 2012;186:999–1007. 22904183 10.1164/rccm.201203-0478OC

[btad335-B10] De Vries M , Oude Munnink BB, Deijs M et al Performance of VIDISCA-454 in feces-suspensions and serum. Viruses 2012;4:1328–34. 23012629 10.3390/v4081328 PMC3446766

[btad335-B11] Ding C , He X, Simon HD. On the equivalence of nonnegative matrix factorization and spectral clustering. In: *Proceedings of the 2005 SIAM International Conference on Data Mining*. Newport Beach, April 21-23, 2005. Philadelphia: SIAM, 2005, 606–10.

[btad335-B12] Ding C , Li T, Peng W et al Orthogonal nonnegative matrix t-factorizations for clustering. In: *Proceedings of the 12th ACM SIGKDD International Conference on Knowledge Discovery and Data Mining*. Philadelphia, August 20 - 23, 2006. New York: ACM, 2006, 126–35.

[btad335-B13] García C , Tebbji F, Daigneault M et al The human gut microbial metabolome modulates fungal growth via the TOR signaling pathway. mSphere 2017;2:e00555-17. 29242837 10.1128/mSphere.00555-17 PMC5729221

[btad335-B14] Gaudelet T , Malod-Dognin N, Pržulj N. Higher-order molecular organization as a source of biological function. Bioinformatics 2018;34:i944–53. 30423061 10.1093/bioinformatics/bty570 PMC6129285

[btad335-B15] Haak BW , Argelaguet R, Kinsella CM et al Integrative transkingdom analysis of the gut microbiome in antibiotic perturbation and critical illness. mSystems 2021;6:e01148-20. 33727397 10.1128/mSystems.01148-20 PMC8546997

[btad335-B16] He X , Cai D, Niyogi P. Laplacian score for feature selection. Adv Neural Inf Process Syst, Vancouver, December 5 - 8, 2005. Cambridge: MIT, 2005, 1–8.

[btad335-B17] Honda K , Littman DR. The microbiome in infectious disease and inflammation. Annu Rev Immunol 2012;30:759–95. 22224764 10.1146/annurev-immunol-020711-074937 PMC4426968

[btad335-B18] Hsiao J-R , Chang C-C, Lee W-T et al The interplay between oral microbiome, lifestyle factors and genetic polymorphisms in the risk of oral squamous cell carcinoma. Carcinogenesis 2018;39:778–87. 29668903 10.1093/carcin/bgy053

[btad335-B19] Janda JM , Abbott SL. 16S rRNA gene sequencing for bacterial identification in the diagnostic laboratory: pluses, perils, and pitfalls. J Clin Microbiol 2007;45:2761–4. 17626177 10.1128/JCM.01228-07 PMC2045242

[btad335-B20] Jin T , Cao L, Zhang B et al Hypergraph induced convolutional manifold networks. Proceedings of the 28th International Joint Conference on Artificial Intelligence, Macao, August 10 - 16, 2019. Menlo Park: AAAI, 2019, 2670–6.

[btad335-B21] Kaufman L , Rousseeuw PJ. Finding Groups in Data: An Introduction to Cluster Analysis. New Jersey: John Wiley & Sons, 2009.

[btad335-B22] Lee DD , Seung HS. Learning the parts of objects by non-negative matrix factorization. Nature 1999;401:788–91. 10548103 10.1038/44565

[btad335-B23] Lee YJ , Arguello ES, Jenq RR et al Protective factors in the intestinal microbiome against Clostridium difficile infection in recipients of allogeneic hematopoietic stem cell transplantation. J Infect Dis 2017;215:1117–23. 28498996 10.1093/infdis/jix011 PMC5426375

[btad335-B24] Legoff J , Resche-Rigon M, Bouquet J et al The eukaryotic gut virome in hematopoietic stem cell transplantation: new clues in enteric graft-versus-host disease. Nat Med 2017;23:1080–5. 28759053 10.1038/nm.4380

[btad335-B25] Li T , Ding C. The relationships among various nonnegative matrix factorization methods for clustering. In: *Sixth International Conference on Data Mining (ICDM'06)*, Hong Kong, December 18-22, 2006. Washington: IEEE, 2006, 362–71.

[btad335-B26] Liu S , Shang X. Hierarchical similarity network fusion for discovering cancer subtypes. In: International Symposium on Bioinformatics Research and Applications, Beingjing China, June 8-11, 2018. Berlin: Springer, 2018, 125–36.

[btad335-B27] Liu Y , Hayes DN, Nobel A et al Statistical significance of clustering for high-dimension, low–sample size data. J Am Stat Assoc 2008;103:1281–93.

[btad335-B28] Ludington WB. Higher-order microbiome interactions and how to find them. Trends Microbiol 2022;30:618–21. 35469711 10.1016/j.tim.2022.03.011

[btad335-B29] Ma Y , Hu X, He T et al Clustering and integrating of heterogeneous microbiome data by joint symmetric nonnegative matrix factorization with Laplacian regularization. IEEE/ACM Trans Comput Biol Bioinform 2020;17:788–95. 28961122 10.1109/TCBB.2017.2756628

[btad335-B30] Ma Y , Hu X, He T, et al A robust symmetric nonnegative matrix factorization framework for clustering multiple heterogeneous microbiome data. Preprints. org 2017, 2017040105. DOI: 10.20944/preprints201704.0105.v1

[btad335-B31] Mac Aogáin M , Narayana JK, Tiew PY et al Integrative microbiomics in bronchiectasis exacerbations. Nat Med 2021;27:688–99. 33820995 10.1038/s41591-021-01289-7

[btad335-B32] McInnes L , Healy J, Melville J. Umap: uniform manifold approximation and projection for dimension reduction. arXiv, arXiv:1802.03426, 2018.

[btad335-B33] Nguyen LN , Lopes LCL, Cordero RJB et al Sodium butyrate inhibits pathogenic yeast growth and enhances the functions of macrophages. J Antimicrob Chemother 2011;66:2573–80. 21911344 10.1093/jac/dkr358

[btad335-B34] Pfeiffer JK , Virgin HW. Transkingdom control of viral infection and immunity in the mammalian intestine. Science 2016;351:aad5872. 26816384 10.1126/science.aad5872 PMC4751997

[btad335-B35] Richard ML , Sokol H. The gut mycobiota: insights into analysis, environmental interactions and role in gastrointestinal diseases. Nat Rev Gastroenterol Hepatol 2019;16:331–45. 30824884 10.1038/s41575-019-0121-2

[btad335-B36] Rousseeuw PJ. Silhouettes: a graphical aid to the interpretation and validation of cluster analysis. J Comput Appl Math 1987;20:53–65.

[btad335-B38] Sanchez-Gorostiaga A , Bajić D, Osborne ML et al High-order interactions distort the functional landscape of microbial consortia. PLoS Biol 2019;17:e3000550. 31830028 10.1371/journal.pbio.3000550 PMC6932822

[btad335-B39] Santos JM , Embrechts M. On the use of the adjusted rand index as a metric for evaluating supervised classification. In: International Conference on Artificial Neural Networks, Limassol, Cyprus, September 14-17, 2009. Berlin: Springer, 2009, p.175–84.

[btad335-B40] Shkoporov AN , Hill C. Bacteriophages of the human gut: the “known unknown” of the microbiome. Cell Host Microbe 2019;25:195–209. 30763534 10.1016/j.chom.2019.01.017

[btad335-B41] Sokol H , Leducq V, Aschard H et al Fungal microbiota dysbiosis in IBD. Gut 2017;66:1039–48. 26843508 10.1136/gutjnl-2015-310746 PMC5532459

[btad335-B42] Sovran B , Planchais J, Jegou S et al Enterobacteriaceae are essential for the modulation of colitis severity by fungi. Microbiome 2018;6:1–16. 30172257 10.1186/s40168-018-0538-9 PMC6119584

[btad335-B43] Strehl A , Ghosh J. Cluster ensembles–-a knowledge reuse framework for combining multiple partitions. J Mach Learn Res 2002;3:583–617.

[btad335-B44] Vinh NX , Epps J, Bailey J. Information theoretic measures for clusterings comparison: variants, properties, normalization and correction for chance. J Mach Learn Res 2010;11:2837–54.

[btad335-B45] Wagg C , Schlaeppi K, Banerjee S et al Fungal-bacterial diversity and microbiome complexity predict ecosystem functioning. Nat Commun 2019;10:4841. 31649246 10.1038/s41467-019-12798-y PMC6813331

[btad335-B46] Wang B , Mezlini AM, Demir F et al Similarity network fusion for aggregating data types on a genomic scale. Nat Methods 2014;11:333–7. 24464287 10.1038/nmeth.2810

[btad335-B47] Wayne Litaker R , Vandersea MW, Kibler SR et al Recognizing dinoflagellate species using its rDNA sequences 1. J Phycol 2007;43:344–55.

[btad335-B48] Xu T , Le TD, Liu L et al CancerSubtypes: an R/bioconductor package for molecular cancer subtype identification, validation and visualization. Bioinformatics 2017;33:3131–3. 28605519 10.1093/bioinformatics/btx378

[btad335-B49] Zhang Y , Hu X, Jiang X. Multi-view clustering of microbiome samples by robust similarity network fusion and spectral clustering. IEEE/ACM Trans Comput Biol Bioinform 2017;14:264–71. 26513798 10.1109/TCBB.2015.2474387

[btad335-B50] Zhou D , Huang J, Schölkopf B. Learning with hypergraphs: clustering, classification, and embedding. Adv Neural Inf Process Syst, Canada, December 4 - 7, 2006. Cambridge: MIT, 2006, 1–8.

[btad335-B51] Zhu C , Zhang Y, Li YE et al Joint profiling of histone modifications and transcriptome in single cells from mouse brain. Nat Methods 2021;18:283–92. 33589836 10.1038/s41592-021-01060-3 PMC7954905

[btad335-B52] Zuo T , Lu X-J, Zhang Y et al Gut mucosal virome alterations in ulcerative colitis. Gut 2019;68:1169–79. 30842211 10.1136/gutjnl-2018-318131 PMC6582748

