# Supplementary material for: HONMF: integration analysis of multi-omics microbiome data via matrix factorization and hypergraph
Source: Bioinformatics. 2023 May 22;39(6):btad335. doi: 10.1093/bioinformatics/btad335 (PMC10243929; doi:10.1093/bioinformatics/btad335)
Supplement: btad335_Supplementary_Data [file btad335_supplementary_data.docx]

**Additional file 1: Supplementary Methods for “HONMF: integration analysis of multi-omics microbiome data via matrix factorization and hypergraph”**

**Optimization algorithm for HONMF**

In this section, we presented an alternative iteration algorithm to solve the optimization problem of HONMF model.

$$\min_{{H^{\left( i \right)},G}^{\left( i \right)},S} J=\sum_{i=1}^{3} \left\| A^{\left( i \right)}-H^{\left( i \right)}{G^{\left( i \right)}H}^{\left( i \right)^{T}} \right\|_{F}^{2}+\frac{\alpha}{2}\sum_{i=1}^{3} \left\| S-H^{\left( i \right)}H^{\left( i \right)^{T}} \right\|_{F}^{2}+\eta\sum_{i=1}^{3} \left\| H^{\left( i \right)^{T}}H^{\left( i \right)}-I \right\|_{F}^{2}+\beta\left\| S\boldsymbol{1}-\boldsymbol{1} \right\|_{F}^{2}+\gamma\sum_{i=1}^{3} tr\left( H^{\left( i \right)^{T}}L_{hyg}^{(i)}H^{\left( i \right)} \right)$$

$s.t. H^{\left( i \right)},G^{\left( i \right)},S,\alpha,\beta,\gamma,\eta\geq0.$ (1)

Here, $H^{\left( i \right)}\in R_{+}^{n\times k}$ represents the matrix of low-dimensional representation (i.e., latent variables) for the $i\mathrm{th}$ data modality (bacterial, fungal or virus compositional profile) of the samples. $G\in R_{+}^{k\times k}$ represents the connections among different clusters and is symmetric. *S* is the leaned sample-sample similarity matrix that can be used for clustering and data visualization of the microbial samples. $\mathbf{1}$ is a column vector with all elements to be 1s. $L_{hyg}^{(i)}$ represents the hypergraph Laplacian for the $i\mathrm{th}$ data modality, and it capture high-order relationship in original microbiome data[1-3]. $A^{\left( i \right)}$ is the similarity matrix obtained from $i\mathrm{th}$ microbial compositional profile matrix $X^{\left( i \right)}$. $\eta$ is the parameter that reflects strength of the orthogonal constraint imposed to the columns of $H^{\left( i \right)}$, and is set $\eta=10$ for all datasets. $\beta$ is a parameter that is used to control the strength of the constraint $S\mathbf{1}=\mathbf{1}$ and is set $\beta=1$ for all datasets. $\gamma$ is graph regularization parameter. In the later section, we will discuss how to choose $\alpha$ and $\gamma$.

In the objective function, the first term, $\sum_{i=1}^{3} \left\| A^{\left( i \right)}-H^{\left( i \right)}{G^{\left( i \right)}H}^{\left( i \right)^{T}} \right\|_{F}^{2}$, is standard tri-factor symmetric NMF loss function for bacterial, fungal and virus compositional profile data. The sample similarity matrix $A^{\left( i \right)}$ can be obtained by Gaussion kernel function.

$$A_{jl}^{(i)}=exp\left( -\frac{\left\| x_{j}^{(i)} -x_{l}^{(i)} \right\|_{F}^{2}}{\mu{{(\sigma}_{jl}^{(i)})}^{2}} \right), (2)$$

$$\sigma_{jl}^{(i)}=\frac{E_{jl}+mean\left( E\left( j,N_{j} \right) \right)+mean\left( E\left( k,N_{l} \right) \right)}{3}, (3)$$

where $x_{j}^{(i)}$ and $x_{l}^{(i)}$ are the observed data for sample $j$ and sample $l$ in the $i$th composition profile. $\mu$ is a parameter that can be empirically set. We set $\mu=0.5$ for all datasets. $\sigma_{jl}^{(i)}$ is the bandwidth parameter [4]. $E_{jl}$ is the squared Euclidean distance between sample $j$ and $l$. $N_{j}$ is the set of nearest neighbors of the $j\mathrm{th}$ sample. In this study, we set $\left| N_{j} \right|=20$. $mean\left( E\left( j,N_{j} \right) \right)$ is the average of the squared Euclidean distances between the $j\mathrm{th}$ sample and its neighbors.

The second term, $\sum_{i=1}^{3} \left\| S-H^{\left( i \right)}H^{\left( i \right)^{T}} \right\|_{F}^{2}$, is a consensus graph fusion operation that integrates different compositional profile data to learn a sample similarity matrix $S$. One of the advantages is that it regularizes each kernel $H^{\left( i \right)}H^{\left( i \right)^{T}}$ from each compositional data towards a consensus graph $S$. The third term, $\sum_{i=1}^{3} \left\| H^{\left( i \right)^{T}}H^{\left( i \right)}-I \right\|_{F}^{2}$, encourages the low-dimensional representations $H^{\left( i \right)}$ to be column-orthogonal, and is used to preserve the uniqueness of the solution. The fourth term, $\left\| S\boldsymbol{1}-\boldsymbol{1} \right\|_{F}^{2}$ is a normalization term on $S$ that encourages each row in $S$ to have summation close to 1. The last term, $\sum_{i=1}^{3} tr\left( H^{\left( i \right)^{T}}L_{hyg}^{(i)}H^{\left( i \right)} \right)$, is a hypergraph regularized term used to preserve high-order geometrical structure information in original data, which is important to reveal the complex relationships for more than two nodes.

The optimal problem of objective function (Eq.1) can be divided into three sub-problems and be alternately solved.

(1) Fixing $G^{\left( i \right)}$ and $S$, updating $H^{\left( i \right)}$

The optimal problem is independent among distinct modality data. We only consider the terms related to $H^{\left( i \right)}$. The objective reduces to

$$\min_{H^{\left( 1 \right)}} J=\left\| A^{\left( 1 \right)}-H^{\left( 1 \right)}{G^{\left( 1 \right)}H}^{\left( 1 \right)^{T}} \right\|_{F}^{2}+\frac{\alpha}{2}\left\| S-H^{\left( 1 \right)}H^{\left( 1 \right)^{T}} \right\|_{F}^{2}+\eta\left\| H^{\left( 1 \right)^{T}}H^{\left( 1 \right)}-I \right\|_{F}^{2}+\gamma tr\left( H^{\left( i \right)^{T}}L_{hyg}^{(i)}H^{\left( i \right)} \right)$$

$s.t. H^{\left( 1 \right)},\alpha,\gamma,\eta\geq0.$ (4)

Based on trace operation, Eq.4 can be rewritten as the following:

$J_{1}=tr\left( A^{\left( 1 \right)^{T}}A^{\left( 1 \right)}-2A^{\left( 1 \right)^{T}}H^{\left( 1 \right)}G^{\left( 1 \right)}H^{\left( 1 \right)^{T}}+H^{\left( 1 \right)}G^{\left( 1 \right)}H^{\left( 1 \right)^{T}}H^{\left( 1 \right)}G^{\left( 1 \right)}H^{\left( 1 \right)^{T}} \right)+\gamma tr\left( H^{\left( i \right)^{T}}L_{hyg}^{(i)}H^{\left( i \right)} \right)+\eta tr\left( H^{\left( 1 \right)^{T}}H^{\left( 1 \right)}H^{\left( 1 \right)^{T}}H^{\left( 1 \right)}-2H^{\left( 1 \right)^{T}}H^{\left( 1 \right)}I+II^{T} \right)+\frac{\alpha}{2}tr\left( S^{T}S-2S^{T}H^{\left( 1 \right)}H^{\left( 1 \right)^{T}}+H^{\left( 1 \right)}H^{\left( 1 \right)^{T}}H^{\left( 1 \right)}H^{\left( 1 \right)^{T}} \right)$ (5)

Next, Lagrange method and KKT conditions are used to solve the optimal problem above. We can obtain the following updating rule for $H^{\left( 1 \right)}$.

$$H_{ij}^{\left( 1 \right)}⟵H_{ij}^{\left( 1 \right)}\frac{\left( A^{\left( 1 \right)}H^{\left( 1 \right)}G^{\left( 1 \right)}+{\eta H}^{\left( 1 \right)}+\alpha S^{T}H^{\left( 1 \right)}+0.5\gamma W_{hyg}^{(1)}H^{\left( 1 \right)} \right)_{ij}}{\left( H^{\left( 1 \right)}G^{\left( 1 \right)}H^{\left( 1 \right)^{T}}H^{\left( 1 \right)}G^{\left( 1 \right)}+\eta H^{\left( 1 \right)}H^{\left( 1 \right)^{T}}H^{\left( 1 \right)}+\alpha H^{\left( 1 \right)}H^{\left( 1 \right)^{T}}H^{\left( 1 \right)}+0.5\gamma D_{hyg}^{(1)}H^{\left( 1 \right)} \right)_{ij}}.(6)$$

Similarly, the updating rule for $H^{\left( 2 \right)}$ and $H^{\left( 3 \right)}$ can be computed as follows:

$$H_{ij}^{\left( 2 \right)}⟵H_{ij}^{\left( 2 \right)}\frac{\left( A^{\left( 2 \right)}H^{\left( 2 \right)}G^{\left( 2 \right)}+{\eta H}^{\left( 2 \right)}+\alpha S^{T}H^{\left( 2 \right)}+0.5\gamma W_{hyg}^{(2)}H^{\left( 2 \right)} \right)_{ij}}{\left( H^{\left( 2 \right)}G^{\left( 2 \right)}H^{\left( 2 \right)^{T}}H^{\left( 2 \right)}G^{\left( 2 \right)}+\eta H^{\left( 2 \right)}H^{\left( 2 \right)^{T}}H^{\left( 2 \right)}+\alpha H^{\left( 2 \right)}H^{\left( 2 \right)^{T}}H^{\left( 2 \right)}+0.5\gamma D_{hyg}^{(2)}H^{\left( 2 \right)} \right)_{ij}}.(7)$$

$$H_{ij}^{\left( 3 \right)}⟵H_{ij}^{\left( 3 \right)}\frac{\left( A^{\left( 3 \right)}H^{\left( 3 \right)}G^{\left( 3 \right)}+{\eta H}^{\left( 3 \right)}+\alpha S^{T}H^{\left( 3 \right)}+0.5\gamma W_{hyg}^{(3)}H^{\left( 3 \right)} \right)_{ij}}{\left( H^{\left( 3 \right)}G^{\left( 3 \right)}H^{\left( 3 \right)^{T}}H^{\left( 3 \right)}G^{\left( 3 \right)}+\eta H^{\left( 3 \right)}H^{\left( 3 \right)^{T}}H^{\left( 3 \right)}+\alpha H^{\left( 3 \right)}H^{\left( 3 \right)^{T}}H^{\left( 3 \right)}+0.5\gamma D_{hyg}^{(3)}H^{\left( 3 \right)} \right)_{ij}}.(8)$$

Here, $W_{hyg}^{(1)}$, $W_{hyg}^{(2)}$ and $W_{hyg}^{(3)}$ are adjacency matrices according to hypergraph Laplacian $L_{hyg}^{(1)}$, $L_{hyg}^{(2)}$ and $L_{hyg}^{(3)}$. $D_{hyg}^{(1)}$, $D_{hyg}^{(2)}$ and $D_{hyg}^{(3)}$ are degree matrices.

(2) Fixing $H^{\left( i \right)}$ and $G^{\left( i \right)}$, updating $S$

The objective function with respect to $S$ is as the following.

$\min_{S} J_{2}=\frac{\alpha}{2}\sum_{i=1}^{3} \left\| S-H^{\left( i \right)}H^{\left( i \right)^{T}} \right\|_{F}^{2}+\beta\left\| S\boldsymbol{1}-\boldsymbol{1} \right\|_{F}^{2}$. (9)

$$s.t. S\geq0.$$

The objective function can be rewritten as the following:

$J_{2}=\frac{\alpha}{2}\left( tr\left( S^{T}S-2{S^{T}H}^{\left( 1 \right)}H^{\left( 1 \right)^{T}}+H^{\left( 1 \right)}H^{\left( 1 \right)^{T}}H^{\left( 1 \right)}H^{\left( 1 \right)^{T}} \right)+tr\left( S^{T}S-2S^{T}H^{\left( 2 \right)}H^{\left( 2 \right)^{T}}+H^{\left( 2 \right)}H^{\left( 2 \right)^{T}}H^{\left( 2 \right)}H^{\left( 2 \right)^{T}} \right)+tr\left( S^{T}S-2S^{T}H^{\left( 3 \right)}H^{\left( 3 \right)^{T}}+H^{\left( 3 \right)}H^{\left( 3 \right)^{T}}H^{\left( 3 \right)}H^{\left( 3 \right)^{T}} \right) \right)+\beta tr(e^{T}S^{T}se-2e^{T}S^{T}e+e^{T}e).$(10)

where $e$ is a column vector with all elements being 1s.

By using Lagrange method and KKT conditions, the updating rule for *S* can be obtained as following.

$$S_{ij}⟵S_{ij}\frac{\left( \alpha\sum_{l=1}^{3} H^{\left( l \right)}H^{\left( l \right)^{T}}+2\beta{ee}^{T} \right)_{ij}}{\left( 3\alpha S+2\beta S{ee}^{T} \right)_{ij}}. (11)$$

(3) Fixing $H^{\left( i \right)}$ and $S$, updating $G^{\left( i \right)}$

The objective function with respect to $G^{\left( 1 \right)}$ is as the following.

$$\min_{G^{\left( 1 \right)}} J=\left\| A^{\left( 1 \right)}-H^{\left( 1 \right)}{G^{\left( 1 \right)}H}^{\left( 1 \right)^{T}} \right\|_{F}^{2}.(12)$$

$s.t. G^{\left( i \right)}\geq0.$

Similarity, the updating rule for $G^{\left( 1 \right)}$, $G^{\left( 2 \right)}$and $G^{\left( 3 \right)}$ are computed as:

$$G_{ij}^{\left( 1 \right)}⟵G_{ij}^{\left( 1 \right)}\frac{\left( H^{\left( 1 \right)^{T}}A^{\left( 1 \right)}H^{\left( 1 \right)} \right)_{ij}}{\left( H^{\left( 1 \right)^{T}}H^{\left( 1 \right)}{G^{\left( 1 \right)}H}^{\left( 1 \right)^{T}}H^{\left( 1 \right)} \right)_{ij}}. (13)$$

$$G_{ij}^{\left( 2 \right)}⟵G_{ij}^{\left( 2 \right)}\frac{\left( H^{\left( 3 \right)^{T}}A^{\left( 2 \right)}H^{\left( 2 \right)} \right)_{ij}}{\left( H^{\left( 2 \right)^{T}}H^{\left( 2 \right)}{G^{\left( 2 \right)}H}^{\left( 2 \right)^{T}}H^{\left( 2 \right)} \right)_{ij}}. (14)$$

$$G_{ij}^{\left( 3 \right)}⟵G_{ij}^{\left( 3 \right)}\frac{\left( H^{\left( 3 \right)^{T}}A^{\left( 3 \right)}H^{\left( 3 \right)} \right)_{ij}}{\left( H^{\left( 3 \right)^{T}}H^{\left( 3 \right)}{G^{\left( 3 \right)}H}^{\left( 3 \right)^{T}}H^{\left( 3 \right)} \right)_{ij}}. (15)$$

**Setting the weights for hyperedges**

Given dada matrix *X* and the incidence matrix *H* corresponding to hypergraph, we compute the weight for each hyperedge *e* as following:

$$w\left( e \right)=\sum_{i\in e} \sum_{j\in e} W_{ij} (16)$$

$$W_{ij}=\exp\left( -\frac{\left\| X_{i}-X_{j} \right\|_{2}^{2}}{\sigma} \right) (17)$$

$$\sigma=\frac{\sum_{k\in e} \sum_{l\in e} \left\| X_{k}-X_{l} \right\|_{2}^{2}}{\delta\left( e \right)*\left( \delta\left( e \right)-1 \right)} (18)$$

where $\delta\left( e \right)$ denotes the degree of hyperedge.

**The robustness analysis of HONMF on the hyperparameters**

To test the robustness of HONMF on the hyperparameters, we varied α and γ in the range $\left\{ \alpha^{*}/10,\alpha^{*}/5, \alpha^{*}/2,\alpha^{*}, 2\alpha^{*},5\alpha^{*}, 10\alpha^{*}, \right\}$ and$\left\{ \gamma^{*}/10,\gamma^{*}/5,\gamma^{*}/2,\gamma^{*}, 2\gamma^{*},5\gamma^{*}, 10\gamma^{*} \right\}$, respectively. Here $\alpha^{*}$ and $\gamma^{*}$ are the hyperparameters chosen by the rule in the manuscript. The results are presented in Supplementary Figure S3. For the gut microbiome data, the performance is more stable when the hyperparameter γ varies. The performance is less stable when the hyperparameter α becomes large. One of possible reasons is that large α values enforce the learned sample similarity matrix *S* to be consistent with the inner-product of low-dimension representations of samples. This constraint is too strict, and it results in weak learning ability of HONMF.

**Supplementary Figures and Tables**


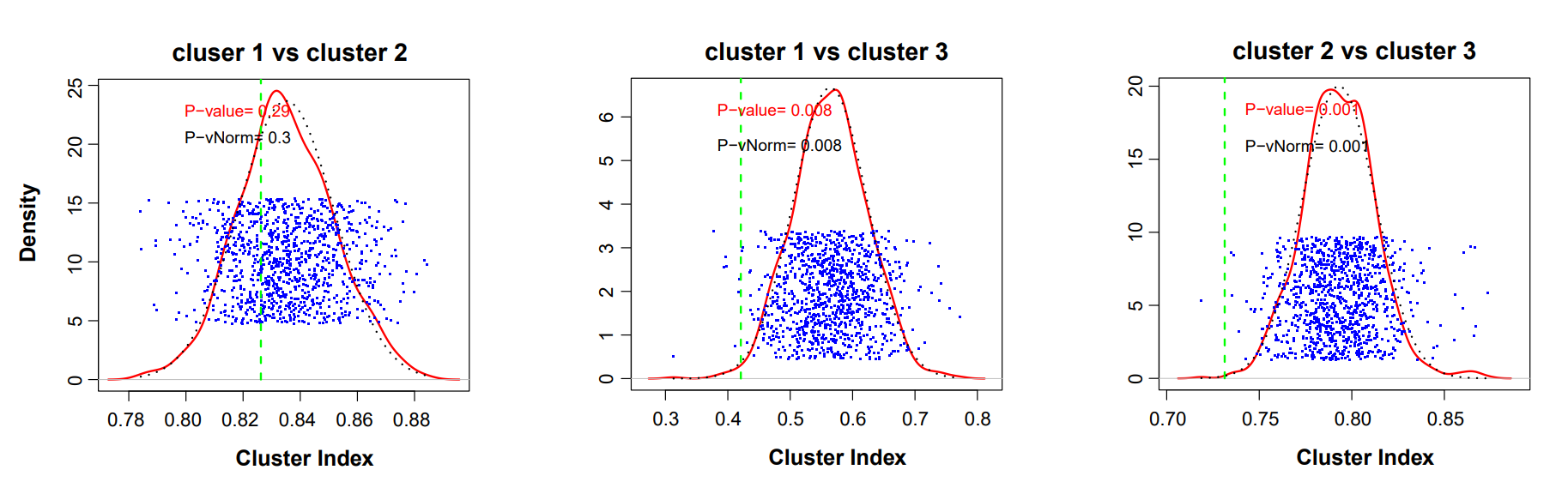


**Supplementary Figure S1:** Assessment of clustering significance on sputum microbiome data. The blue points represent the simulated CIs (cluster indices). The red solid line and black dotted line correspond to the estimated nonparametric density and Gaussian density fit to the simulated CIs[5].


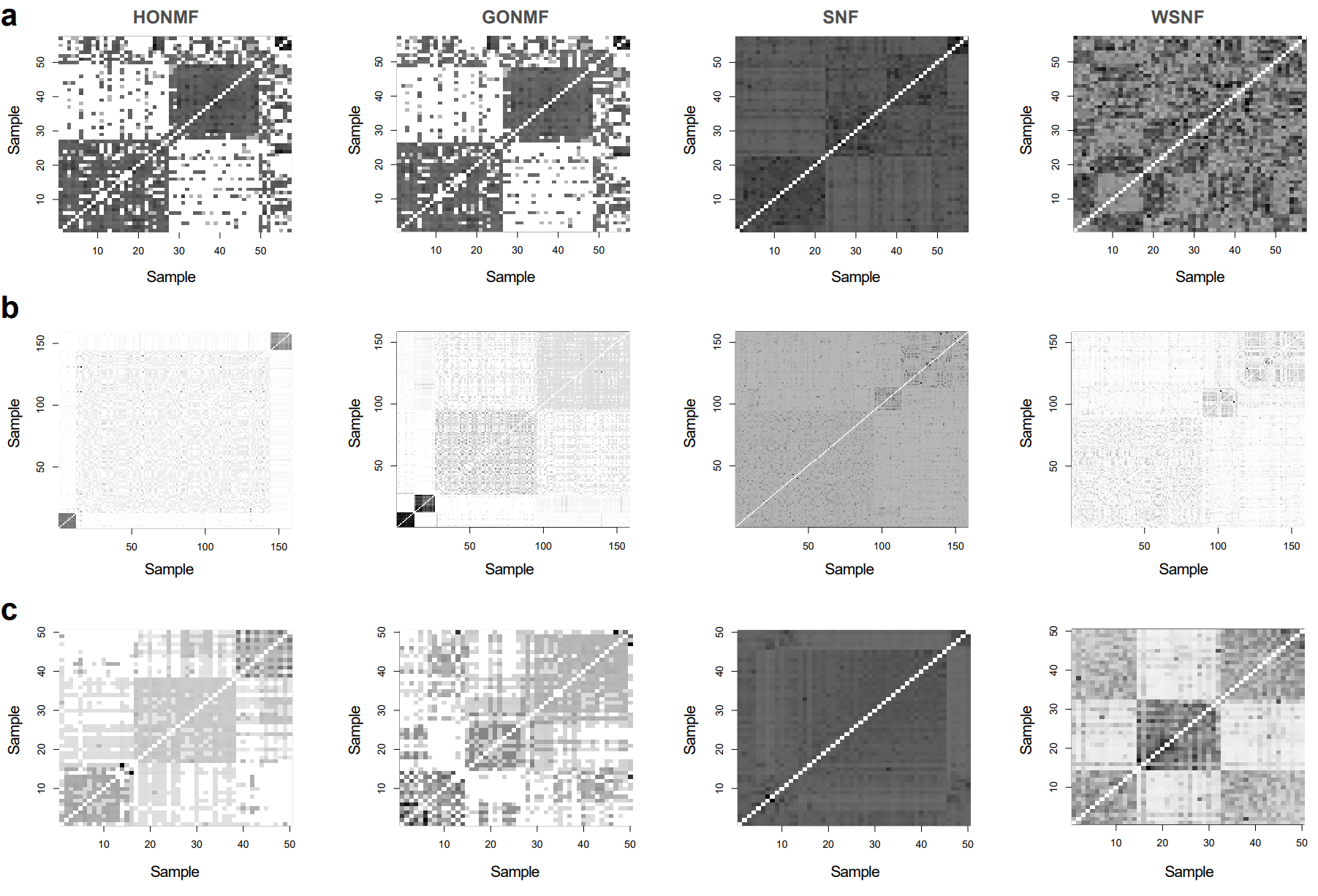


**Supplementary Figure S2:** Heatmaps of sample-sample similarity matrices based on the inferred similarity matrix *S* by HONMF and other competitive methods. a) Heatmaps of sample-sample similarity matrices for gut data. b) Heatmap of sample-sample similarity matrices for sputum data. c) Heatmaps of sample-sample similarity matrices for soil data.


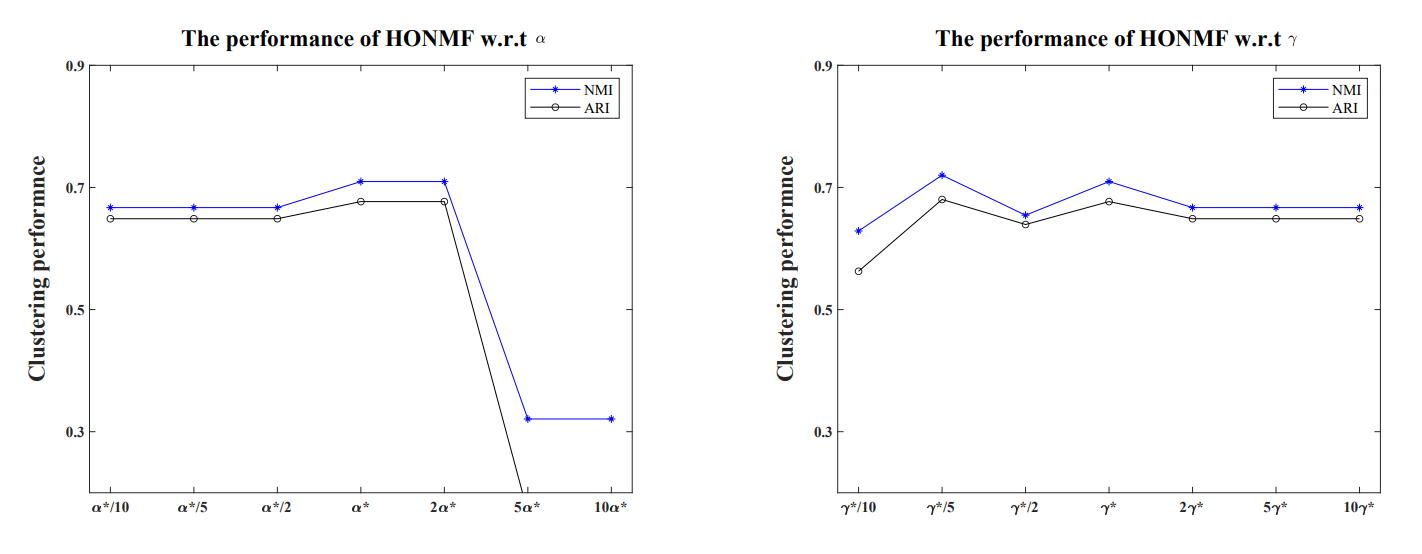


**Supplementary Figure S3:** The robustness analysis of HONMF on the α and γ. Here α* and γ* are the hyperparameters chosen by the rules in our manuscript. We use gut microbiome data and sample types provided in the original publication to test the performance of HONMF.


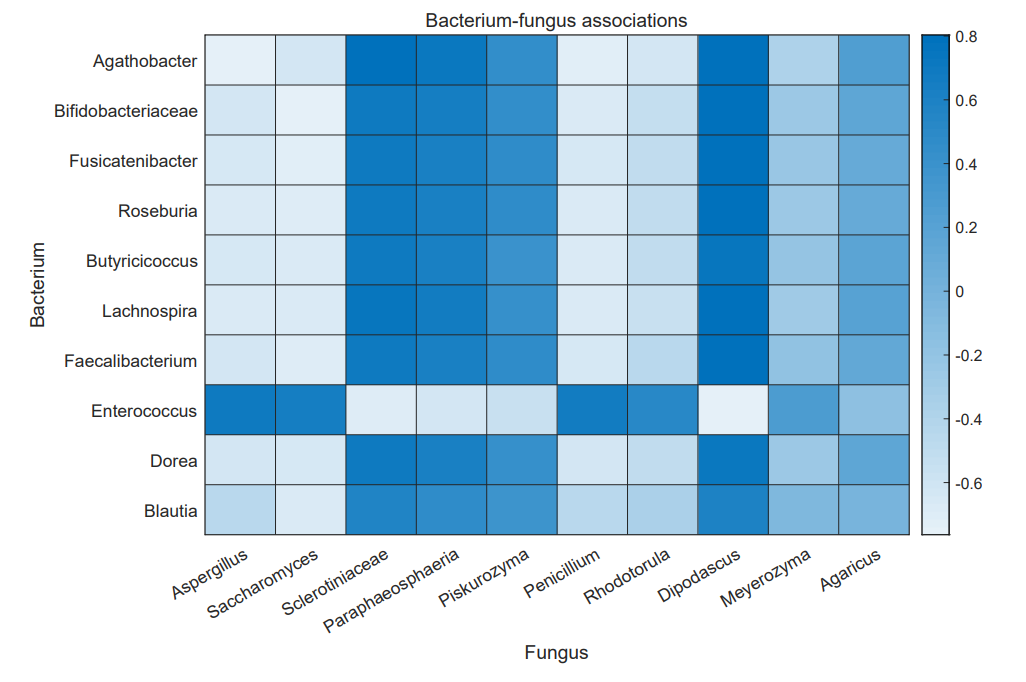


**Supplementary Figure S4:** The heatmap of bacterium-fungus interaction matrix on gut dataset. Bacterium-fungus associations are computed by using the correlation coefficients between discriminative bacterial and fungal species.

**Supplementary Table S1:** The clustering performance for each method on three microbial multi-modal datasets

| Methods | Gut | | Sputum | Soil |
| --- | --- | --- | --- | --- |
|  | ARI | NMI | Silhouette | Silhouette |
| SNF | 0.5932 | 0.6743 | 0.0761 | 0.0120 |
| WSNF | 0.0773 | 0.1813 | 0.4783 | 0.3830 |
| MOFA+ | 0.5048 | 0.5975 | 0.6132 | -0.2428 |
| HONMF | 0.6786 | 0.7096 | 0.7469 | 0.4713 |

**Supplementary Table S2**: The performance of HONMF by conducting ablation experiments

|  | Gut | | Sputum | Soil |
| --- | --- | --- | --- | --- |
|  | ARI | NMI | Silhouette | Silhouette |
| $\alpha=0$ | 0.5432 | 0.5722 | 0.4291 | 0.3882 |
| $\gamma=0$ | 0.4047 | 0.4231 | 0.6234 | 0.5249 |
| $\eta=0$ | 0.0318 | 0.1197 | 0.8049 | 0.1081 |
| HONMF | 0.6786 | 0.7096 | 0.7469 | 0.4713 |

**Supplementary Table S3**: A comparison between the Laplacian graph version of HONMF (GONMF) and hypergraph version of HONMF.

|  | Gut | | | Sputum | Soil |
| --- | --- | --- | --- | --- | --- |
|  | ARI | NMI | Silhouette | Silhouette | Silhouette |
| GONMF | 0.6355 | 0.6506 | 0.2475 | 0.6021 | 0.1236 |
| HONMF | 0.6786 | 0.7096 | 0.3421 | 0.7469 | 0.4713 |

**Supplementary Table S4** The statistical information of three datasets

| Data | (#) Features | (#) Samples | (#) Views |
| --- | --- | --- | --- |
| Gut | Bacteria: 180  Fungi: 18  Viruses: 42 | 57 | 3 |
| Sputum | Bacteria: 992  Fungi: 16  Viruses: 703 | 158 | 3 |
| Soil | Bacteria: 2461  Fungi: 365 | 50 | 2 |

**Supplementary Table S5** The performance of HONMF in term of different dimension of factor *H*

| Factors | Gut | | | Sputum | Soil |
| --- | --- | --- | --- | --- | --- |
|  | NMI | ARI | Silhouette | Silhouette | Silhouette |
| 2 | 0.6649 | 0.6593 | 0.2449 | 0.7690 | 0.4605 |
| 3 | 0.6076 | 0.5824 | 0.3804 | 0.7469 | 0.4713 |
| 4 | 0.7096 | 0.6786 | 0.3421 | 0.7656 | 0.5271 |
| 5 | 0.6361 | 0.6008 | 0.1455 | 0.7562 | 0.5530 |
| HONMF | 0.7096 | 0.6786 | 0.3421 | 0.7469 | 0.4713 |

**References**

[1] Gaudelet T, Malod-Dognin N, Pržulj N. Higher-order molecular organization as a source of biological function. Bioinformatics, 2018, 34: i944-i953

[2] Jin T, Cao L, Zhang B*, et al.* Hypergraph Induced Convolutional Manifold Networks. City: Year. 2670-2676

[3] Zhou D, Huang J, Schölkopf B. Learning with hypergraphs: Clustering, classification, and embedding. Advances in neural information processing systems, 2006, 19:

[4] Wang B, Mezlini A M, Demir F*, et al.* Similarity network fusion for aggregating data types on a genomic scale. Nature methods, 2014, 11: 333-337

[5] Liu Y, Hayes D N, Nobel A*, et al.* Statistical significance of clustering for high-dimension, low–sample size data. Journal of the American Statistical Association, 2008, 103: 1281-1293
